# Impaired BK_Ca_ channel function in native vascular smooth muscle from humans with type 2 diabetes

**DOI:** 10.1038/s41598-017-14565-9

**Published:** 2017-10-25

**Authors:** Madeline Nieves-Cintrón, Arsalan U. Syed, Olivia R. Buonarati, Robert R. Rigor, Matthew A. Nystoriak, Debapriya Ghosh, Kent C. Sasse, Sean M. Ward, Luis F. Santana, Johannes W. Hell, Manuel F. Navedo

**Affiliations:** 1 Department of Pharmacology, University of California, Davis, CA 95616 USA; 2 Diabetes and Obesity Center, Department of Medicine, University of Louisville, Louisville, KY 40202 USA; 3 Sasse Surgical Associates, Reno, NV 89502 USA; 4 Department of Physiology and Cell Biology, University of Nevada, Reno School of Medicine, Reno, NV 89557 USA; 5 Department of Physiology & Membrane Biology, University of California, Davis, CA 95616 USA

## Abstract

Large-conductance Ca^2+^-activated potassium (BK_Ca_) channels are key determinants of vascular smooth muscle excitability. Impaired BK_Ca_ channel function through remodeling of BK_Ca_ β1 expression and function contributes to vascular complications in animal models of diabetes. Yet, whether similar alterations occur in native vascular smooth muscle from humans with type 2 diabetes is unclear. In this study, we evaluated BK_Ca_ function in vascular smooth muscle from small resistance adipose arteries of non-diabetic and clinically diagnosed type 2 diabetic patients. We found that BK_Ca_ channel activity opposes pressure-induced constriction in human small resistance adipose arteries, and this is compromised in arteries from diabetic patients. Consistent with impairment of BK_Ca_ channel function, the amplitude and frequency of spontaneous BK_Ca_ currents, but not Ca^2+^ sparks were lower in cells from diabetic patients. BK_Ca_ channels in diabetic cells exhibited reduced Ca^2+^ sensitivity, single-channel open probability and tamoxifen sensitivity. These effects were associated with decreased functional coupling between BK_Ca_ α and β1 subunits, but no change in total protein abundance. Overall, results suggest impairment in BK_Ca_ channel function in vascular smooth muscle from diabetic patients through unique mechanisms, which may contribute to vascular complications in humans with type 2 diabetes.

## Introduction

The World Health Organization estimates that ~350 million people worldwide have non-insulin-dependent type 2 diabetes, a number that is expected to double by 2030^1^. Vascular complications (e.g. hypertension, coronary heart disease, stroke) are among the most prominent causes of morbidity and mortality in type 2 diabetic patients^1–3^. While endothelial dysfunction has long been recognized as a key link in the pathogenesis of vascular complications during diabetes, emerging data in human and animal models also implicate vascular smooth muscle dysfunction in this process^3–11^. At present however, there is limited information about the mechanisms underlying changes in vascular smooth muscle function in native cells from type 2 diabetic patients.

The contractile state of vascular smooth muscle in small resistance arteries is controlled by multiple ion channels^12^. Among them, large-conductance Ca^2+^-activated potassium (BK_Ca_) channels play a key role in control of vascular smooth muscle contractility via tonic regulation of membrane potential^13^. Physiologically, these channels are activated by membrane depolarization and localized Ca^2+^ release events through ryanodine receptors (e.g. Ca^2+^ sparks) located in the sarcoplasmic reticulum^13–15^. The opening of BK_Ca_ channels by Ca^2+^ sparks results in the occurrence of BK_Ca_-mediated spontaneous transient outward currents (STOCs), leading to vascular smooth muscle hyperpolarization and consequently vasodilation. Conversely, their inhibition causes membrane potential depolarization and vasoconstriction. BK_Ca_ channels are composed of four pore-forming α subunits in association with accessory β subunits^16^. Four distinct BK_Ca_ β subunits (β1-β4) have been identified, with BK_Ca_ β1 being predominantly expressed in vascular smooth muscle^16^. The BK_Ca_ β1 subunit regulates BK_Ca_ channel activity by modulating its apparent Ca^2+^ sensitivity as well as its biophysical properties^17^. Impairment in BK_Ca_ β1 expression and function has been implicated in aberrant vascular BK_Ca_ channel activity in animal models of diabetes and hypertension, and in immortalized cell lines from human diabetic subjects^9,18–25^. However, whether similar alterations in BK_Ca_ channel activity occur in native, freshly dissociated vascular smooth muscle cells from humans with type 2 diabetes has not been established.

Here, we investigated BK_Ca_ channel function in freshly isolated, small resistance adipose arteries and corresponding native vascular smooth muscle cells from obese non-diabetic and clinically diagnosed type 2 diabetic patients. Data revealed a reduction in iberiotoxin (IbTx) sensitivity of intact arteries from diabetic patients. This was associated with impaired BK_Ca_ channel activity due to decreased BK_Ca_ β1 function in diabetic vascular smooth muscle cells. In contrast to results observed in animal models^9,21–23,25^, impaired BK_Ca_ β1 function was not due to changes in BK_Ca_ β1 abundance, but to a reduced functional association between BK_Ca_ α and β1 subunits in diabetic cells. Thus, results suggest a mechanism for altered vascular BK_Ca_ channel function in patients with type 2 diabetes with similar, but not identical features to those observed in animal models of diabetes.

## Results

To establish whether BK_Ca_ channel function is impaired in native vascular smooth muscle cells during type 2 diabetes, small diameter arteries were dissected from adipose tissue obtained from obese non-diabetic and clinically diagnosed type 2 diabetic patients undergoing surgical sleeve gastrectomy (see Methods section). Available patient information is included in Supplementary Table S1.

### Decreased IBTx sensitivity in arteries from diabetic subjects

**Figure 1:**
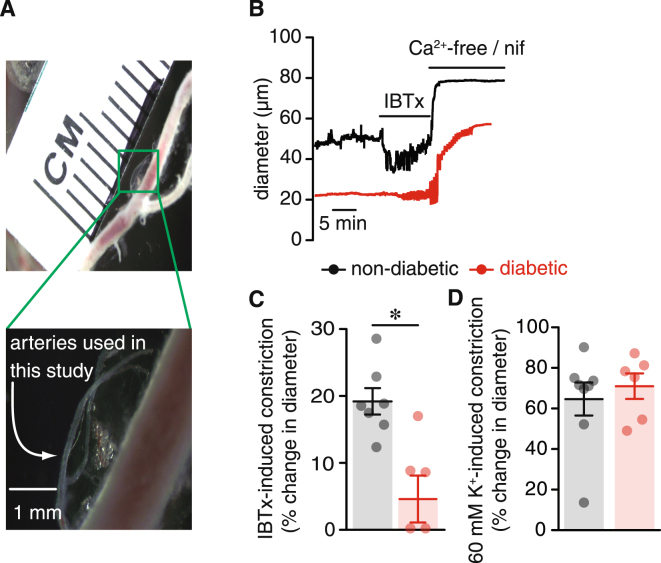
Adipose arteries from diabetic subjects exhibit blunted response to IBTx. (**A**) Exemplary image of small resistance adipose arteries used in this study. The image below shows an expanded view of the area in the green square. (**B)** Representative diameter recordings of pressurized (80 mmHg) adipose arteries from non-diabetic and diabetic patients before and after perfusion of 100 nM iberiotoxin or a Ca^2+^ free + nifedipine (1 μM) solution. (**C)** Summary of iberiotoxin-induced constriction in non-diabetic (n = 7 arteries, 4 subjects) and diabetic (n = 5 arteries, 5 subjects) adipose arteries pressurized to 80 mmHg. (**D)** Scatter/bar plot summarizing vascular tone at 80 mmHg in response to 60 mM extracellular K^+^ in non-diabetic (n = 8 arteries, 4 subjects) and diabetic (n = 6 arteries, 5 subjects) adipose arteries. *P < 0.05, Mann-Whitney test. Significance was compared between non-diabetic and diabetic datasets.

Small diameter adipose arteries (average passive diameter between 50–90 μm; see Fig. 1A) from non-diabetic and clinically diagnosed diabetic patients were pressurized to 80 mmHg, and allowed to develop stable myogenic tone. Mean basal myogenic tone was 44 ± 9% in non-diabetic arteries, and was modestly, although not significantly, elevated to 49 ± 3% in arteries from diabetic patients (*P* = 0.325). To assess the contribution of BK_Ca_ channels to vascular function, adipose arteries from non-diabetic and diabetic patients were challenged with the selective BK_Ca_ channel inhibitor iberiotoxin (IBTx; 100 nM). IBTx caused robust vasoconstriction in non-diabetic arteries (24% decrease in diameter) (Fig. 1B and C; Supplementary Table S2). This is consistent with BK_Ca_ channel activity opposing vasoconstriction. In contrast, IBTx had little effect on pressurized adipose arteries from diabetic patients (4% decrease in diameter; Fig. 1B and C; Supplementary Table S2; *P* < 0.05). Arteries from both groups responded to external application of a 60 mM K^+^ solution with robust constriction (Fig. 1D; Supplementary Table S2), suggesting that differences in the IBTx response were not simply due to the inability of diabetic arteries to respond to a contractile stimulus. This decrease in IBTx sensitivity in diabetic arteries suggests impairment in BK_Ca_ channel function in arteries from diabetic patients.

### Decreased STOC amplitude and frequency in vascular smooth muscle from diabetic patients

**Figure 2:**
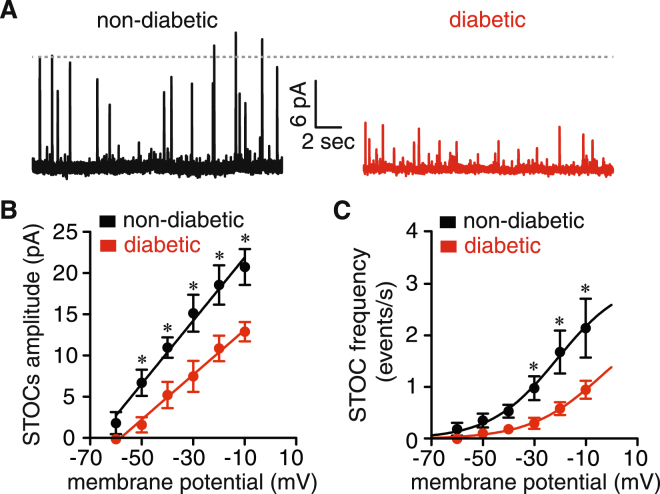
STOCs amplitude and frequency are reduced in vascular smooth muscle from diabetic patients. (**A**) Representative traces of spontaneous whole-cell BK_Ca_ currents (e.g. STOCs) at −40 mV in vascular smooth muscle from non-diabetic and diabetic patients. (**B**,**C)** Voltage dependency of STOC amplitude (**B**) and frequency (**C**) in cells from non-diabetic (n = 10 cells, 4 subjects) and diabetic (n = 13 cells, 4 subjects) patients. Solid lines represent the best fit of the amplitude data using a linear regression function and of the frequency data using a Boltzmann sigmoidal function. *P < 0.05, unpaired *t* test. Significance was compared between corresponding non-diabetic and diabetic datasets.

BK_Ca_-mediated STOCs were recorded at membrane potential ranging from −60 mV to −10 mV. Figure 2A displays representative STOCs recordings from freshly dissociated vascular smooth muscle obtained from small diameter adipose arteries from non-diabetic and diabetic patients at −40 mV. STOCs amplitude and frequency increased with membrane depolarization in cells of both groups. However, a significant reduction in STOC amplitude was evident at several membrane potentials examined in cells from diabetic compared to non-diabetic patients (Fig. 2A and B). The slope of the STOC amplitude-voltage relationship was significantly smaller in diabetic (0.27 ± 0.01 pA/mV) compared to non-diabetic cells (0.38 ± 0.02 pA/mV; *P* < 0.05; *F* test) (Fig. 1B). STOC frequency was also significantly decreased in diabetic vascular smooth muscle cells at voltages from −30 to −10 mV (Fig. 1C; *P* < 0.05). The voltage dependency of STOC frequency was shifted toward more depolarized potentials in diabetic cells (V_50_ = −4 ± 4 mV) compared to non-diabetic cells (V_50_ = −21 ± 7 mV; Fig. 2C). These results suggest that BK_Ca_ channel activity is altered in vascular smooth muscle from diabetic patients.

### Ca^2+^ spark activity is similar in vascular smooth muscle from non-diabetic and diabetic patients

**Figure 3:**
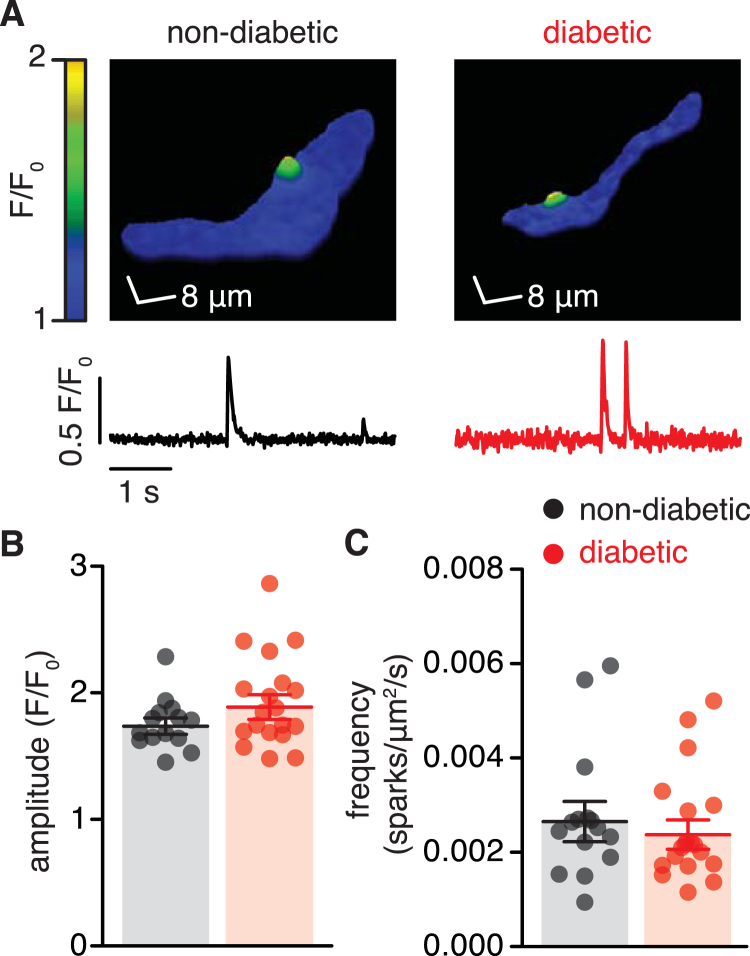
The frequency and amplitude of Ca^2+^ sparks is similar in non-diabetic and diabetic vascular smooth muscle. (**A)** Representative three-dimensional pseudo-color images of Ca^2+^ sparks (*upper panels*) and fractional fluorescence traces (F/F_0_; *lower panels*) from those sites in fluo-4 AM-loaded vascular smooth muscle cells from non-diabetic and diabetic patients. Scatter/bar plots summarizing Ca^2+^ spark amplitude (**B**) and frequency (**C**) in cells from non-diabetic (n = 254 events, 14 cells, 3 subjects) and diabetic (n = 333 events, 19 cells, 5 subjects) patients. *P < 0.05, Mann-Whitney test. Significance was compared between non-diabetic and diabetic datasets.

STOC activity in vascular smooth muscle is tightly coupled to Ca^2+^ sparks^13,15,26^. These events were recorded from non-diabetic and diabetic vascular smooth muscle loaded with the fluorescent Ca^2+^ indicator fluo-4 AM to evaluate whether changes in Ca^2+^ spark activity could account for impairment in BK_Ca_ channel function in vascular smooth muscle from diabetic patients (Fig. 3A). Figure 3B and C shows that the amplitude and frequency of Ca^2+^ sparks were similar in non-diabetic and diabetic cells. These results suggest that impaired BK_Ca_ channel function is not due to changes in Ca^2+^ spark activity in vascular smooth muscle from diabetic patients.

### Impaired BK_Ca_ activity in vascular smooth muscle from diabetic patients

**Figure 4:**
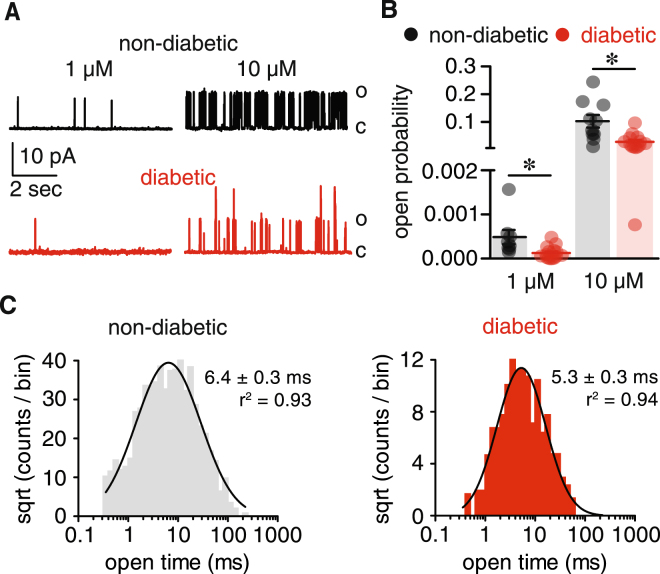
Decreased BK_Ca_ channel activity in vascular smooth muscle from diabetic patients. (**A**) Representative single BK_Ca_ channel traces at −40 mV from excised membrane patches of isolated human non-diabetic and diabetic vascular smooth muscle cells in the presence of 1 μM and 10 μM free Ca^2+^ bath solution (C: closed; O: open). (**B)** Scatter/bar plot summarizing BK_Ca_ channel open probability (P_o_) at the indicated free Ca^2+^ concentration from human non-diabetic (n = 18 cells, 9 subjects) and diabetic (n = 27 cells, 15 subjects) cells. *P < 0.05, Mann-Whitney test. (**C)** Open dwell time histograms of BK_Ca_ channels in non-diabetic and diabetic vascular smooth muscle. The goodness of the fit was assessed with R^2^ and the *F* test was used for comparison between open time fits. Significance was compared between non-diabetic and diabetic datasets.

Single BK_Ca_ currents were recorded in excised membrane patches to evaluate whether differences in the functional properties of BK_Ca_ channels account for compromised channel activity during diabetes. BK_Ca_ currents were monitored at a physiological membrane potential (−40 mV) with bath solution containing either 1 μM or 10 μM free Ca^2+^. Increasing free Ca^2+^ from 1 μM to 10 μM augmented the open probability (*P*_*o*_) of BK_Ca_ channels in both non-diabetic and diabetic cells (Fig. 4A and B). However, BK_Ca_ channel *P*_*o*_ was significantly lower in diabetic cells compared to non-diabetic cells at each Ca^2+^ concentration tested (Fig. 4A and B). In addition, open time histograms revealed a shift toward shorter openings in diabetic cells (Fig. 4C; *P* < 0.05; *F* test). These results suggest a reduction in apparent Ca^2+^ sensitivity and dwell open time of BK_Ca_ channels in vascular smooth muscle cells from diabetic patients.

### Altered BK_Ca_ β1 subunit function in vascular smooth muscle from diabetic patients

**Figure 5:**
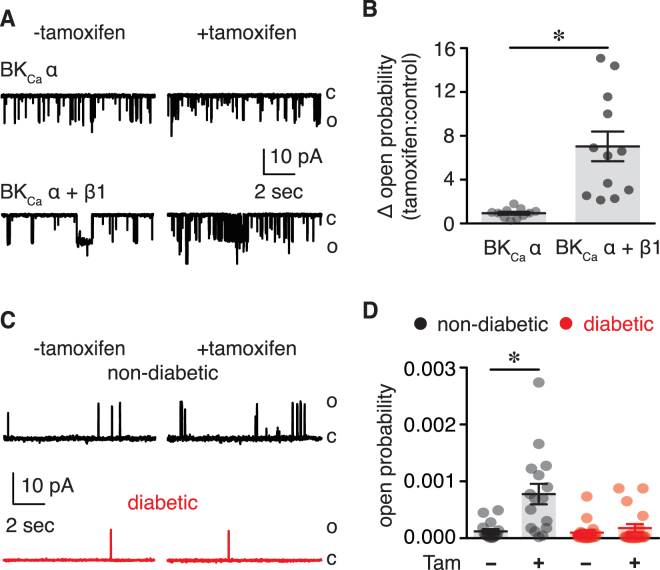
Impaired BK_Ca_ β1 subunit function in human diabetic vascular smooth muscle. (**A**) Representative single BK_Ca_ channel records at +40 mV and 1 μM free Ca^2+^ obtained from excised membrane patches of HEK293 cells expressing only BK_Ca_ α subunit or coexpressing BK_Ca_ α + BK_Ca_ β1 subunits in the absence and presence of tamoxifen (1 μM). (**B**) Bar plot summarizing the change in open probability (tamoxifen: control) for HEK293 cells expressing BK_Ca_ α (n = 11 cells) or BK_Ca_ α + BK_Ca_ β1 (n = 12 cells) subunits. *P < 0.05, unpaired *t* test **C)** Representative single BK_Ca_ channel traces recorded from excised membrane patches of vascular smooth muscle from non-diabetic and diabetic patients with 1 μM free Ca^2+^ in the bath before (−) and after (+) application of 1 μM tamoxifen. (**D)** Amalgamated data summarizing BK_Ca_ open probability in non-diabetic (n = 17 cells, 9 subjects) and diabetic (n = 19 cells, 8 subjects) cells in the presence or absence of tamoxifen. (C: closed; O: open) *P < 0.05, Wilcoxon test. Significance for Fig. 5B was compared between cells co-expressing BK_Ca_ α + BK_Ca_ β1 vs. BK_Ca_ α only. Significance for Fig. 5D was compared to corresponding - tamoxifen datasets.

The BK_Ca_ channel function is highly dependent on expression and function of the accessory BK_Ca_ β subunit^17,27^. To examine whether impaired BK_Ca_ channel activity in vascular smooth muscle from diabetic patients is due to changes in BK_Ca_ β subunit function, single BK_Ca_ currents were recorded in the absence or presence of tamoxifen (1 μM). This drug increases BK_Ca_ channel *P*_*o*_ by engaging the BK_Ca_ β subunit as shown in Fig. 5A and B, and as previously reported by our group and others^9,19,28^. Whereas tamoxifen significantly increased the *P*_*o*_ of BK_Ca_ in non-diabetic cells, it had a minimal effect in vascular smooth muscle from diabetic subjects (Fig. 5C and D). These results suggest that a reduction in BK_Ca_ β subunit function may contribute to impaired BK_Ca_ channel activity in vascular smooth muscle cells from diabetic patients.

### Reduced association between BK_Ca_ α and BK_Ca_ β1 subunits in vascular smooth muscle from diabetic patients

**Figure 6:**
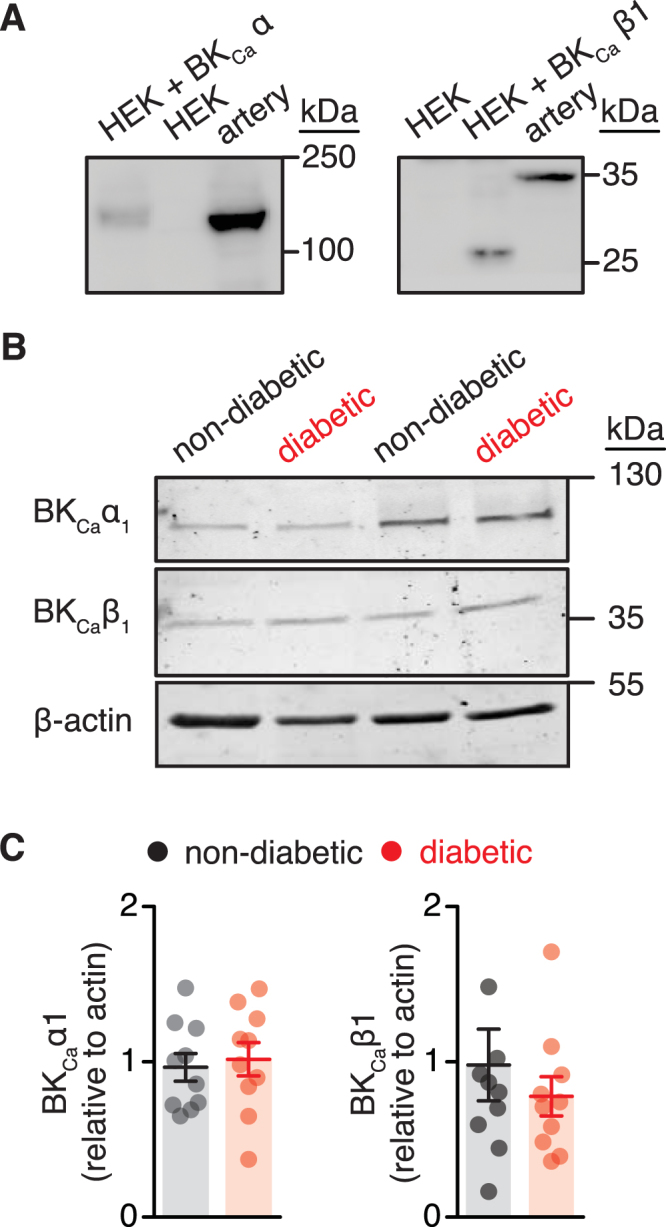
No change in BK_Ca_ subunit total protein levels in arterial lysates from non-diabetic and diabetic patients. (**A**) Representative immunoreactive bands corresponding to BK_Ca_ α1 and BK_Ca_ β1 subunits in lysates from untransfected HEK293 cells (HEK), HEK293 cells transfected with either BK_Ca_ α1 or BK_Ca_ β1 subunit and human arteries from non-diabetic patients (n = 3 lysates per condition). Full-length blots are shown in Supplementary Fig. S2A. (**B**) Representative immunoreactive bands corresponding to BK_Ca_ α1 and BK_Ca_ β1 subunits, and β-actin as a normalization control. Full-length blots are shown in Supplementary Fig. S2B. (**C**) Corresponding densitometric summary data for each subunit obtained using arterial lysates from non-diabetic (n = 10 lysates) and diabetic (n = 10 lysates) patients. *P < 0.05, Mann-Whitney test. Significance was compared between non-diabetic and diabetic datasets.

To examine the molecular mechanisms underlying altered BK_Ca_ channel activity and BK_Ca_ β subunit function during diabetes, protein expression of the pore forming BK_Ca_ α subunit and BK_Ca_ β1 subunit were examined using Western blot analysis. Antibodies were validated for specificity using HEK293 cells expressing either BK_Ca_ α or BK_Ca_ β1 subunits (Fig. 6A and Supplementary Fig. S1). Bands of similar molecular weight for the BK_Ca_ α subunit were found in lysates from HEK293 cells stably expressing this subunit and human adipose arteries (Fig. 6A). The molecular weight of the BK_Ca_ β1 subunit was slightly higher in lysates from human adipose arteries compared to HEK293 cell transfected with a BK_Ca_ β1 construct (Fig. 6A), perhaps reflecting differences in post-translational modifications as recently observed^29^. A non-specific band of ~40 kDa was observed in lysates from HEK cells, but only cells expressing BK_Ca_ β1 showed the expected immunoreactive band at 25 kDa (Fig. 6A and Supplementary Figs S1A and S2A). Also note that only one immunoreactive band of expected molecular weight for the BK_Ca_ β1 was detected in human adipose arterial lysates (Supplementary Figs S1B and S2A). Subsequent examination of total protein abundance for BK_Ca_ α and BK_Ca_ β1 subunits in human non-diabetic and diabetic arteries revealed no differences between groups (Fig. 6B and C).

**Figure 7:**
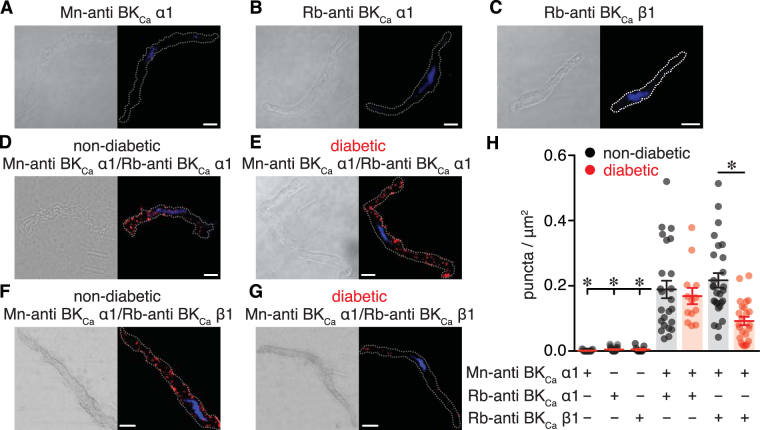
Decreased association between BK_Ca_ α1 and BK_Ca_ β1 subunits in vascular smooth muscle cells from diabetic patients. (**A–C**) Differential interference contrast (DIC) and confocal fluorescent PLA puncta (red) and DAPI (blue) images of freshly dissociated human vascular smooth muscle labeled with mouse-anti BK_Ca_ α1 (**A**), rabbit-anti BK_Ca_ α1 (**B**), and rabbit-anti BK_Ca_ β1 (**C**) antibodies. (**D,E)** DIC (left) and fluorescence PLA (red)/DAPI (blue) (right) images of dissociated vascular smooth muscle from non-diabetic (**D**) and diabetic (**E**) patients co-labeled with two distinct antibodies for the BK_Ca_ α1 subunit. (**F**,**G**) DIC (left) and fluorescence PLA (red)/DAPI (blue) (right) images of dissociated human vascular smooth muscle from non-diabetic (**F**) and diabetic (**G**) patients co-labeled for BK_Ca_ α1 and BK_Ca_ β1 subunits. Scale bar = 10 μm. (**H**) Quantification of PLA fluorescent puncta per μm^2^ cell area for non-diabetic and diabetic vascular smooth muscle cells labeled for mouse-anti BK_Ca_ α1 (n = 19 cells), rabbit-anti BK_Ca_ α1 (n = 27 cells), rabbit-anti BK_Ca_ β1 (n = 12 cells), mouse-anti BK_Ca_ α1 + rabbit-anti BK_Ca_ α1 (n = 24 non-diabetic, 14 diabetic cells); mouse-anti BK_Ca_ α1 + rabbit-anti BK_Ca_ β1 (n = 27 non-diabetic, 24 diabetic cells). *P < 0.05, Mann-Whitney test. Significance for columns 1, 2, 3 and 5 was compared to column 4, and column 7 was compared to column 6.

To further explore mechanisms that may contribute to compromised BK_Ca_ channel activity and BK_Ca_ β1 subunit function during diabetes, the coupling between BK_Ca_ α and BK_Ca_ β1 was examined using Proximity Ligation Assay (PLA)^30^ and immunofluorescence analysis. PLA fluorescent puncta are generated when proteins of interest are 40 nm or less apart. PLA signals were negligible when primary antibodies for BK_Ca_ α and/or BK_Ca_ β1 subunits were omitted from the preparation (Fig. 7A,B,C and H). As a positive control, vascular smooth muscle from non-diabetic and diabetic subjects stained with two different BK_Ca_ α subunit antibodies demonstrated robust PLA signals with similar number of PLA puncta (Fig. 7D,E and H). In vascular smooth muscle co-labeled for BK_Ca_ α and BK_Ca_ β1, data revealed a significant reduction in PLA puncta in diabetic cells compared to non-diabetic cells (Fig. 7F,G and H). Immunofluorescence analysis showed strong BK_Ca_ α-associated immunofluorescence of similar intensity (normalized to cytosol) along the plasma membrane of non-diabetic (1.7 ± 0.1) and diabetic (1.6 ± 0.1) vascular smooth muscle (*P* = 0.460; Mann Whitney Test; Supplementary Fig. S3). Conversely, the intensity of the BK_Ca_ β1-associated fluorescence along the plasma membrane was markedly reduced in diabetic cells (0.8 ± 0.1) compared to non-diabetic cells (1.5 ± 0.1; *P* < 0.05; Mann Whitney Test; Supplementary Fig. S3). Altogether, these results suggest that BK_Ca_ β1-mediated regulation of BK_Ca_ channels may be compromised in vascular smooth muscle from adipose arteries of diabetic patients due to suppressed functional coupling between BK_Ca_ α and BK_Ca_ β1 subunits, rather than a reduction in cellular subunit abundance.

## Discussion

In this study we report three major novel findings related to BK_Ca_ channels in native small diameter adipose arteries and vascular smooth muscle cells from non-diabetic and type 2 diabetic patients. First, BK_Ca_ channel activity acts to oppose pressure-induced constriction in isolated human resistance adipose arteries, but this is compromised in arteries from diabetic patients. Second, BK_Ca_ channel activity is impaired in vascular smooth muscle from diabetic patients, as reflected by reduced apparent Ca^2+^ sensitivity, dwell open time and BK_Ca_ β subunit function. Third, compromised BK_Ca_ β subunit function during diabetes is not associated with changes in total protein abundance of this subunit, but rather seems to be the result of a reduction in the association/coupling between BK_Ca_ α and BK_Ca_ β1 subunits. The implications of these changes are significant as they may impact vascular reactivity and/or contribute to vascular complications in humans with type 2 diabetes, independent of changes in endothelial function^6,24,31–33^.

Results of experiments examining the effects of IbTx on arterial tone suggest that BK_Ca_ channels serve an important physiological role to oppose pressure-induced vasoconstriction in human adipose arteries, but become severely compromised in adipose arteries from diabetic patients (Fig. 1). These results suggest that BK_Ca_ channel function is compromised in adipose arteries from humans with diabetes. Consistent with this, BK_Ca_-mediated STOCs amplitude and frequency as well as single BK_Ca_ channel function were significantly reduced in dissociated vascular smooth muscle from adipose arteries from diabetic subjects (Figs 2 and 4). Similar results were observed in a number of studies using vascular smooth muscle from several vascular beds and different animal models of diabetes^9,21–23,25,34^. A reduction in BK_Ca_ channel activity and BK_Ca_ β1 function may also contribute to vascular complications by aggravating vessel wall remodeling and fibrosis during diabetes^35^. Thus, a potential common BK_Ca_-mediated mechanism contributing to vascular complications during diabetes may be engaged in a variety of species. In animal models of diabetes, alterations in BK_Ca_ channel function have been associated, in part, to changes in the activity of Ca^2+^ sparks^21,22,25^. Here, no changes in Ca^2+^ sparks properties were detected between dissociated vascular smooth muscle from adipose arteries from non-diabetic and diabetic patients (Fig. 3). These results are similar to those found in vascular smooth muscle from high fat diet mice in which Ca^2+^ spark activity was unaffected compared to cells from low fat fed mice^9^. Discrepancies with other studies could arise from experimental conditions, use of smooth muscle from different vascular beds, or intrinsic differences between murine and human cells/tissue^31,36^. Regardless, our results implicate other mechanisms as culprit for comprised BK_Ca_ channel activity in human vascular smooth muscle during diabetes.

The accessory BK_Ca_ β1 subunit finely tunes the activity of BK_Ca_ channels in vascular smooth muscle by increasing their voltage- and calcium-sensitivity^27^. Impaired BK_Ca_ channel function has been associated with suppression of BK_Ca_ β1 subunit function in several studies with animal models of diabetes^9,21–23,25^. Moreover, compromised BK_Ca_ channel activity was linked with suppression of BK_Ca_ β1 subunit function in human vascular smooth muscle from Han Chinese patients with hypertension^24^. We found that BK_Ca_ β1 subunit function was significantly suppressed in diabetic vascular smooth muscle from adipose tissue as assessed by tamoxifen sensitivity (Figs 4 and 5). Note that consistent with experiments here (Fig. 5), several groups including ours have validated and confirmed that the effects of tamoxifen on BK_Ca_ channels require the presence of the BK_Ca_ β1 subunit^9,19,28^. The impairment in BK_Ca_ β1 subunit function helps explain the reduced apparent Ca^2+^ sensitivity and dwell open time of BK_Ca_ channels as well as the blunted IBTx-induced constriction in diabetic compared to non-diabetic vascular smooth muscle cells/adipose arteries. The loss of BK_Ca_ β1 subunit function in animal models of diabetes^9,21–23,25^ and in humans with hypertension^24^ has been mainly attributed to transcriptional and/or post-translational changes in BK_Ca_ β1 subunit expression. In contrast with these seemingly consistent molecular mechanisms, no changes in total BK_Ca_ α and BK_Ca_ β1 protein levels were detected between non-diabetic and diabetic arterial lysates (Fig. 6). Rather, PLA data revealed a reduced association between BK_Ca_ α and BK_Ca_ β1 subunits in diabetic cells compared to non-diabetic cells (Fig. 7). Furthermore, immunofluorescence imaging provided evidence of a decreased surface expression of BK_Ca_ β1, but not BK_Ca_ α, in diabetic cells compared to non-diabetic cells (Supplementary Fig. S2). This is important as recent studies have suggested that dynamic trafficking of the BK_Ca_ β1 subunit to the plasma membrane is essential for coupling with the mostly membrane bound, pore forming BK_Ca_ α subunit in vascular smooth muscle^37,38^. This dynamic interaction between BK_Ca_ subunits contributes to vascular reactivity under basal conditions^37^ and in response to chronic angiotensin II signaling^38^. Also note that a recent study using human vascular smooth muscle has shown that changes in K_V_1.5 surface expression (perhaps due to altered trafficking of the subunit) rather than differences in total protein levels has been associated with impaired K_V_1.5 channel and vascular reactivity in humans with coronary artery disease^39^. Thus, it is tempting to speculate, for future studies, that a change in BK_Ca_ β1 subunit trafficking may contribute to compromised BK_Ca_ channel activity and vascular reactivity in humans with diabetes.

Vascular complications during diabetes may also be associated with impairment in the function of other ion channels in vascular smooth muscle. For example, it was recently reported that L-type Ca^2+^ channel (LTCC) activity was significantly augmented in vascular smooth muscle from humans with diabetes^40^. This can stimulate Ca^2+^ influx to increase global intracellular Ca^2+^ in vascular smooth muscle. Interestingly, use of a mathematical model of rodent vascular smooth muscle electrophysiology and Ca^2+^ dynamics showed that increased LTCC activity predominantly contributes to impaired intracellular Ca^2+^ during diabetic hyperglycemia^41^. Yet, the model also revealed that this effect on global intracellular Ca^2+^ is substantially amplified when an increase in LTCC activity is accompanied by a decrease in the activity of potassium (K^+^) channels^41^. Indeed, previous studies have shown a selective suppression in voltage-gated K^+^ (K_V_) and BK_Ca_ channel activity that is associated with downregulation of K_V_2.1 and BK_Ca_ β1 subunit, respectively, in vascular smooth muscle from diabetic mice on a high fat diet^9,10^. Thus, potentiation of LTCC activation and suppression of K^+^ channel activity, including that of BK_Ca_ channels as in this study, may synergize to impact vascular reactivity and/or contribute to vascular complications in humans with diabetes. Given the role of K_V_ channels in vascular smooth muscle excitability due to their influence on membrane potential, it will be important to examine the function of these channels as well as the individual contributions of different K_V_ subunits in human cells during control conditions and in diabetes. Collectively, these data will critically inform the development of novel computational models specific to human vascular smooth muscle. This computational approach may help identify and predict the relative contribution of many elements (i.e. LTCC, K_V_ channels, BK_Ca_ channels, etc.) that interact non-linearly to control vascular smooth muscle excitability in humans during physiological and pathological conditions.

The use of human tissue from obese non-diabetic and clinically diagnosed type 2 diabetic patients provides unparalleled translational significance. Yet, factors such as underlying environmental factors, biological variables such as sex and age, genetic background, disease progression, prescription history, personal habits (e.g. smoking) and comorbidities may confound results. Despite these limitations, data in this study are internally consistent and reproducible across different experimental approaches. Indeed, we also recently demonstrated high reproducibility in biochemical and electrophysiological outcomes using similar tissue samples^40^. Furthermore, whereas the use of tissue from obese non-diabetic patients is less than ideal as a true “control”, it does highlight the added stress imposed by diabetes in mediating alterations in BK_Ca_ channel activity. To conclude, and to the best of our knowledge, this study provides the first direct evidence that compromised BK_Ca_ channel activity in native, freshly dissociated vascular smooth muscle may contribute to vascular complications in type 2 diabetic patients. Interestingly, the mechanisms underlying aberrant vascular BK_Ca_ channel activity in humans with diabetes follow some, but not all, the molecular features observed in animal models of diabetes. Gathering information from native human cells may be necessary for development of rational strategies to treat vascular complications in humans with type 2 diabetes.

## Methods

### Human Tissue (Study Approval)

Excised adipose arteries from obese patients undergoing surgical sleeve gastrectomy and that were either non-diabetic or clinically diagnosed with type 2 diabetes were used. Samples were obtained after Institutional Review Board (IRB) approval from the University of Nevada Reno School of Medicine (IRB ID: 2013-019) and in accordance with the guidelines of the *Declaration of Helsinki*. The need for informed consent was waived by IRBs at the University of Nevada Reno School of Medicine (IRB ID: 2013-019) and the University of California Davis School of Medicine (IRB ID: 597267-1) because the tissue is considered “waste”, has no codification that could be used to identify patients and was determined not to be human subject research in accordance with United States of America federal regulations, as defined by 45 CFR 46.102(f). This precludes the acquisition of detailed clinical profiles other than sex, age and whether the patient was diabetic or not (see Table S1). Therefore, no exclusions were made due to medication history or presence of comorbidities. Patients were considered diabetic if they had a hemoglobin A-1c level equal to or greater than 6.5% on pre-surgical testing, or if they were on medication for diabetes treatment^42^. Collected tissue was placed in cold phosphate-buffered saline (PBS) solution containing (in mM): 138 NaCl, 3 KCl, 10 Na_2_HPO_4_, 2 NaH_2_PO_4_, 5 D-glucose, 0.1 CaCl_2_, and 0.1 MgSO_4_, pH 7.4 with NaOH until used.

### Vascular Smooth Muscle Cell Isolation

Single vascular smooth muscle cells were dissociated from small diameter adipose arteries from non-diabetic and type 2 diabetic patients using enzymatic digestion as previously described^40^. Adipose arteries were dissected in ice-cold dissection buffer composed of (in mM): 140 NaCl, 5 KCl, 2 MgCl_2_, 10 D-glucose, and 10 HEPES, pH 7.4 with NaOH. Following dissection, arteries were cut in small pieces and digested in dissection buffer supplemented with papain (26 U/ml) and dithiothreitol (1 mg/mL) at 37 °C for 15 minutes. After this first incubation period, the solution was exchanged with dissection buffer supplemented with collagenase type H (1.95 U/mL), elastase (0.5 mg/mL) and soybean trypsin Inhibitor (1 mg/ml) at 37 °C for 15 minutes. Cells were then washed in ice-cold dissection buffer three times. Glass pipettes of decreasing diameters were then used to gently triturate arteries and obtain single vascular smooth muscle cells. Isolated cells were maintained in ice-cold dissection buffer until use.

### Arterial Diameter Measurements

Freshly dissected small diameter adipose arteries were cannulated on glass micropipettes and mounted in a 5 mL myograph chamber (Living Systems Instrumentation, St. Albans, VT) as described previously^9,43,44^. The vessels were pressurized to 20 mmHg and allowed to equilibrate while continuously perfused (37 °C, 30 min, 3–5 mL/min) with physiological saline solution (PSS) consisting of (in mM): 119 NaCl, 4.7 KCl, 2 CaCl_2_, 24 NaHCO_3_, 1.2 KH_2_PO_4_, 1.2 MgSO_4_, 0.023 ethylenediaminetetraacetic acid (EDTA) and 10 D-glucose aerated with 5% CO_2_/95% O_2_, pH 7.35–7.40. Following an equilibration period, arteries were treated with 60 mM KCl (<5 min; isosmotic replacement of NaCl with KCl) to test their viability. Only arteries with ≥50% constriction in response to 60 mM KCl were used for subsequent experiments. Intravascular pressure was increased to 80 mmHg and arteries were allowed to develop myogenic tone. Lumen diameters of the adipose arteries were recorded using IonOptix software (IonOptix LLC, Westwood-MA). To evaluate the impact of BK_Ca_ channels on myogenic tone, arteries were treated with 100 nM Iberiotoxin (IBTx) (BK_Ca_ channel inhibitor). Percent change in diameter was calculated using the following equation: % myogenic tone = [(DP − DA)/DP] × 100, where DP = passive diameter of the artery in Ca^2+^-free PSS containing the L-type Ca^2+^ channel (LTCC) inhibitor nifedipine (10 μM) and DA = active diameter of the artery in Ca^2+^-containing PSS. Percent constriction in the presence of IBTx or 60 mM K^+^ was calculated using the following equation: % constriction = [(DP − DT)/DP] × 100, where DT = diameter of the artery in Ca^2+^-containing PSS with 100 nM IBTx or 60 mM K^+^ and DP = passive diameter of the artery in Ca^2+^-free PSS containing 10 μM of the LTCC inhibitor nifedipine.

### Electrophysiology

STOCs resulting from the concerted opening of multiple BK_Ca_ channels were recorded at different membrane potentials from freshly dissociated vascular smooth muscle from small diameter adipose arteries using the perforated whole-cell configuration of the patch-clamp technique with an Axopatch 200B amplifier (Molecular Devices, Sunnyvale, CA). Currents were sampled at 10 kHz and low-pass filtered at 2 kHz. The pipette solution consisted of (in mM): 110 K-aspartate, 30 KCl, 10 NaCl, 1 MgCl_2_, 0.5 EGTA, and 10 HEPES, pH adjusted to 7.3 with KOH. The pipette solution was supplemented with 250 μg/ml of amphotericin B (Sigma, St. Louis, MO). Bath solution consisted of (in mM): 130 NaCl, 5 KCl, 2 CaCl_2_, 1 MgCl_2_, 10 glucose and 10 HEPES, pH adjusted to 7.4 with NaOH. STOCs were analyzed using the threshold detection algorithm in Clampfit 10 (Axon Instruments, Inc).

Single BK_Ca_ channel currents were recorded from inside-out membrane patches obtained from freshly dissociated vascular smooth muscle cells from adipose arteries. In some experiments, HEK293 cells expressing either the BK_Ca_ α subunit and EGFP or co-transfected with BK_Ca_ α + β1 subunit in a 1:1 mix and EGFP using PolyPlus jetPRIME were used as previously described^9^. Bath and pipette solutions consisted of (in mM) 140 KCl, 1 HEDTA, 10 HEPES, pH adjusted to 7.3 with Tris. Bath solution was supplemented with CaCl_2_ to achieve the desired free Ca^2+^ concentration, as determined with the MaxChelator software. Single-channel currents were amplified, low-pass filtered at 1 kHz, and sampled at 20 kHz with the Axopatch 200B amplifier using a DigiData 1440 A acquisition board and pClamp 10 software (Axon Instruments, Inc). Currents were elicited by holding the inside-out patch at the specified voltage. Data were directly stored in a PC hard drive. The half-amplitude algorithm of Clampfit 10 was used to detect single-channel openings and to analyze data of channel activity (e.g. open probability; P_o_). The number of BK_Ca_ channels per patch was estimated by holding the patch at +80 mV in the presence of 10 μM free Ca^2+^, which maximizes the P_o_ of these channels^45^. Open time histograms were fit using a log-normal function:

$$y=A\cdot {e}^{\frac{-\{{[\mathrm{ln}(x)-\mathrm{ln}(\tau )]}^{2}\}}{2{\sigma }^{2}}}$$

where A is a constant, τ is the time constant, and σ is the standard deviations of τ. This analysis was validated by using an Akaike’s Information Criterion, which determines the probability that a data set could be described by a particular set of competing models^46^.

### Ca^2+^ Imaging and Analysis

Freshly dissociated vascular smooth muscle cells from small diameter adipose arteries were loaded with the fluorscent Ca^2+^ indicator fluo-4 AM (5 μM) and imaged using an Andor spinning disk confocal microscope system coupled to an Olympus iX81 inverted microscope equipped with a 60x oil immersion lens (numerical aperture 1.49). Andor IQ software was used for acquisition. Images were acquired at 100–120 Hz. Analysis was performed using custom software (SparkLab) written in LabVIEW that employs a computer algorithm as previously described^9,47^.

### Proximity Ligation Assay (PLA)

The Duolink *in situ* PLA detection kit was used to determine colocalization between BK_Ca_ α and BK_Ca_ β1 subunits as previously described^40^. Freshly dissociated myocytes were plated on glass coverslips and allowed to sit for 30 minutes at room temperature. Cells were then fixed in 4% paraformaldehyde (20 min) and quenched in 100 mM glycine (15 min), followed by 3 min washes (2x) in a phosphate-buffered saline (PBS) solution containing (in mM) 138 NaCl, 3 KCl, 10 Na_2_HPO_4_, 2 NaH_2_PO_4_, 5 D-glucose, 0.1 CaCl_2_ and 0.1 MgSO_4_, pH adjusted to 7.4 with NaOH. Cells were then permeabilized for 20 minutes in 0.1% Triton-100 solution in PBS and blocked in Duolink Blocking Solution for 1 hour at 37 °C in a humidity chamber. Cells were incubated overnight with a specific combination of primary antibodies. Mouse anti-BK_Ca_ α (Antibodies Inc; 75–022 1:200) was combined with rabbit anti-BK_Ca_ β1 (Abcam; ab3587; 1:200) antibody. As a positive control, cells were stained with mouse anti-BK_Ca_ α (Antibodies Inc; 75-022 1:200) and rabbit anti-BK_Ca_ α (Alamone; APC-021; 1:200). For negative controls, cells were incubated with only one primary antibody. Antibodies were diluted in Duolink Antibody Diluent Solution per manufacturer instructions. Secondary antibodies containing PLA probes (anti-mouse minus and anti-rabbit plus) were added to the preparation and allowed to incubate for 1 h at 37 °C. A ligation solution consisting of ligase and two distinct oligonucleotides was then added and incubated for 30 minutes at 37 °C. This step was followed by an amplification reaction (100 min, 37 °C), and subsequent washes (2x) for 10 minutes in Duolink Buffer B and 1 × 1 minute in 1% Buffer B per manufacture’s specifications. Coverslips were mounted on a microscope slide with Doulink mounting media. The fluorescence signal was visualized using an Olympus FV1000 confocal system on an Olympus iX81 microscope with a 60X water immersion lens (numerical aperture = 1.4). Images were acquired at different optical planes (z-axis step size = 0.5 μm).

### Immunofluorescence

Immunofluorescence labeling of freshly dissociated vascular smooth muscle from small diameter adipose arteries from non-diabetic and diabetic patients was performed as described previously^10,48^ using a rabbit anti-BK_Ca_ α (Alamone; APC-021; 1:200) and a rabbit anti-BK_Ca_ β1 (Abcam; ab3587; 1:200). The secondary antibody was an Alexa Fluor 568-conjugated donkey anti-rabbit (5 mg/mL) from Molecular Probes. Cells were visualized (512 × 512 pixel images) using an Olympus FV1000 confocal microscope coupled with an Olympus X60 water immersion lens (NA = 1.4) and a zoom of 3.5 (pixel size = 0.1 μm). Images were collected at multiple optical planes (z axis step size = 0.25 μm). The specificity of the primary antibody was tested in negative control experiments in which the primary antibody was substituted with PBS. Cells for each group were imaged using the same laser power, gain settings and pinhole for all treatments.

### Western Blot Analysis

Whole tissue homogenates were prepared from dissected adipose arteries. In some experiments, protein lysates were obtained from HEK293 cells expressing either the BK_Ca_ α or BK_Ca_ β1 subunit or cells that have not been transfected. Samples were flash frozen in liquid nitrogen and homogenized on ice with lysis buffer containing (in mM) 150 NaCl, 10 Na_2_HPO_4_, 1 EDTA with 1% deoxycholic acid, 0.1% sodium dodecyl sulfate, 40 β-glycerophosphate, 20 Na pyrophosphate, 30 NaF, 1 dithiothreitol and protease inhibitors (Complete Mini protease inhibitor cocktail, Roche, San Francisco, CA) in a glass dounce homogenizer. Whole lysates were sonicated for 1 min, rested on ice for 20 min, and centrifuged (5000 rpm, 4 °C) to yield clarified supernatant. Equal amounts of protein (approximately 10 µg) were separated under reducing conditions on a 4–20% gradient polyacrylamide gel according to the manufacturer’s specifications (Bio-Rad, Hercules, CA). Proteins were transferred to polyvinylidene difluoride (PVDF) or nitrocellulose membranes for Western immunoblotting using enhanced chemiluminescence (ECL) and X-ray film or using an Odyssey scanner (LI-COR; Lincoln, NE, USA) with far red or near-infrared (NIR) labeled secondary antibodies. For the Odyssey scanner, nitrocellulose membranes were blocked with 50% Odyssey blocking buffer (LI-COR; Lincoln, NE, USA) (40 min) in Tris-buffered saline (TBS), then incubated with BK_Ca_ subunit specific primary antibodies on a rotating platform for 18–24 h (4 °C). Primary antibodies were rabbit anti-BK_Ca_ α (Alamone; APC-021; 1:500), rabbit anti-BK_Ca_ β1 (Genetex; GTX105666; 1:5000; or Abcam; ab3587; 1:500), anti-BK_Ca_ γ (Alamone; APC-021; 1:500), and mouse anti-β actin (Sigma; AC-40; 1:4000; as a loading control). These antibodies were diluted in TBS with 0.05% tween-20 (t) and 5% Odyssey blocking solution. Membranes were washed 3 times in TBS-t, then incubated (2 h, room temperature) with appropriate secondary antibodies: goat anti-rabbit 680LT (LI-COR; 925-68021; 1:15,000; with 0.1% SDS), 800CW (LI-COR; 925-32211; 1:15,000; for dual staining) or goat anti-mouse 680LT (LI-COR; 925-68020 1:10,000) in TBS-t. For the far red or NIR dyes, band images were acquired using an Odyssey scanner. In some experiments, detection of BK_Ca_ α was acquired by X-ray film. PVDF membranes were blocked with 10% nonfat milk for 1 hour, incubated in rabbit rabbit anti-BK_Ca_ α (Alomone, 1:500) for 2 hours, washed 3 times in TBS-t, incubated in HRP labeled goat anti-mouse (sc-2005; 1:5000; Santa Cruz), then washed 3 times in TBS-t. Bands were identified by ECL and exposure to X-ray film, followed by conventional scanning. Densitometry for immunoreactive bands was performed with ImageJ software (National Institutes of Health). Band quantification was normalized to β actin, and expressed as a percentage of control.

### Chemicals and Statistics

All chemical reagents were from Sigma-Aldrich (St. Louis, MO) unless otherwise stated. Iberiotoxin was from Peptides International (Louisville, KY). Data were analyzed using GraphPad Prism software and expressed as mean ± SEM. Data were assessed for potential outliers using the GraphPad Prism Outlier Test and for normality of distribution using the Shapiro-Wilk or KS normality tests. Statistical significance was then determined using appropriate paired or unpaired Student’s *t*-test, nonparametric tests or One-way analysis of variance (ANOVA) for multiple comparisons with appropriate post hoc test. *P* < 0.05 was considered statistically significant (denoted by * in figures).

### Data Availability

The datasets generated during and/or analyzed during the current study are available from the corresponding author in a reasonable request.

## Electronic supplementary material

Supplementary information
