# Supplementary material for: Impaired BKCa channel function in native vascular smooth muscle from humans with type 2 diabetes
Source: Sci Rep. 2017 Oct 25;7:14058. doi: 10.1038/s41598-017-14565-9 (PMC5656614; doi:10.1038/s41598-017-14565-9)
Supplement: Supplementary file 1 — Supplementary information [file 41598_2017_14565_MOESM1_ESM.pdf]

Impaired BK_{Ca} channel function in native vascular smooth muscle from humans with type 2 diabetes

Madeline Nieves-Cintrón[1]*, Arsalan U. Syed[1], Olivia R. Buonarati[1], Robert R. Rigor[1], Matthew A. Nystoriak[2], Debapriya Ghosh[1], Kent C. Sasse[3], Sean M. Ward[4], Luis F. Santana[5], Johannes W. Hell[1] and Manuel F. Navedo[1]*

[1]Department of Pharmacology, University of California, Davis, CA 95616.
[2]Diabetes and Obesity Center, Department of Medicine, University of Louisville, Louisville, KY 40202.
[3]Sasse Surgical Associates, Reno, NV 89502.
[4]Department of Physiology and Cell Biology, University of Nevada, Reno School of Medicine, Reno, NV 89557.
[5]Department of Physiology & Membrane Biology, University of California, Davis, CA 95616.

*Correspondence to:

Madeline Nieves-Cintrón, PhD
Department of Pharmacology
University of California, Davis
One Shields Avenue, Davis, CA 95616
email: mcnieves@ucdavis.edu
Tel. 530-752-6880
Fax. 530-752-7710

Manuel F. Navedo, PhD
Department of Pharmacology
University of California, Davis
One Shields Avenue, Davis, CA 95616
email: mfnavedo@ucdavis.edu
Tel. 530-752-6880
Fax. 530-752-7710

Supplementary Information

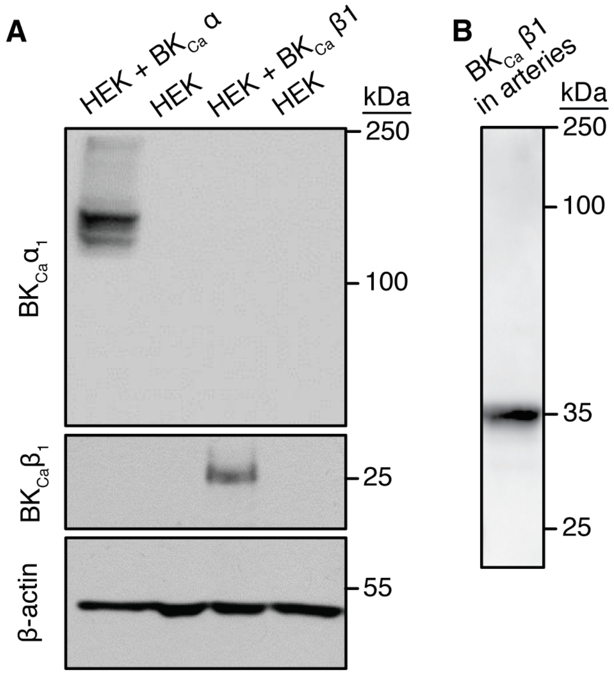

Supplementary Fig. S1: Validation of BK_{Ca} α1 and BK_{Ca} β1 subunit antibodies. A) Representative immunoreactive bands corresponding to BK_{Ca} α1 or BK_{Ca} β1 subunits in lysates from untransfected HEK293 cells, and HEK293 cells transfected with either BK_{Ca} α1 or BK_{Ca} β1 subunit. β-actin was used as loading control (n = 3 lysates per condition). **B)** Representative Western blot showing only one immunoreactive band of the expected molecular weight and corresponding to BK_{Ca} β1 in an arterial lysate from non-diabetic patients.

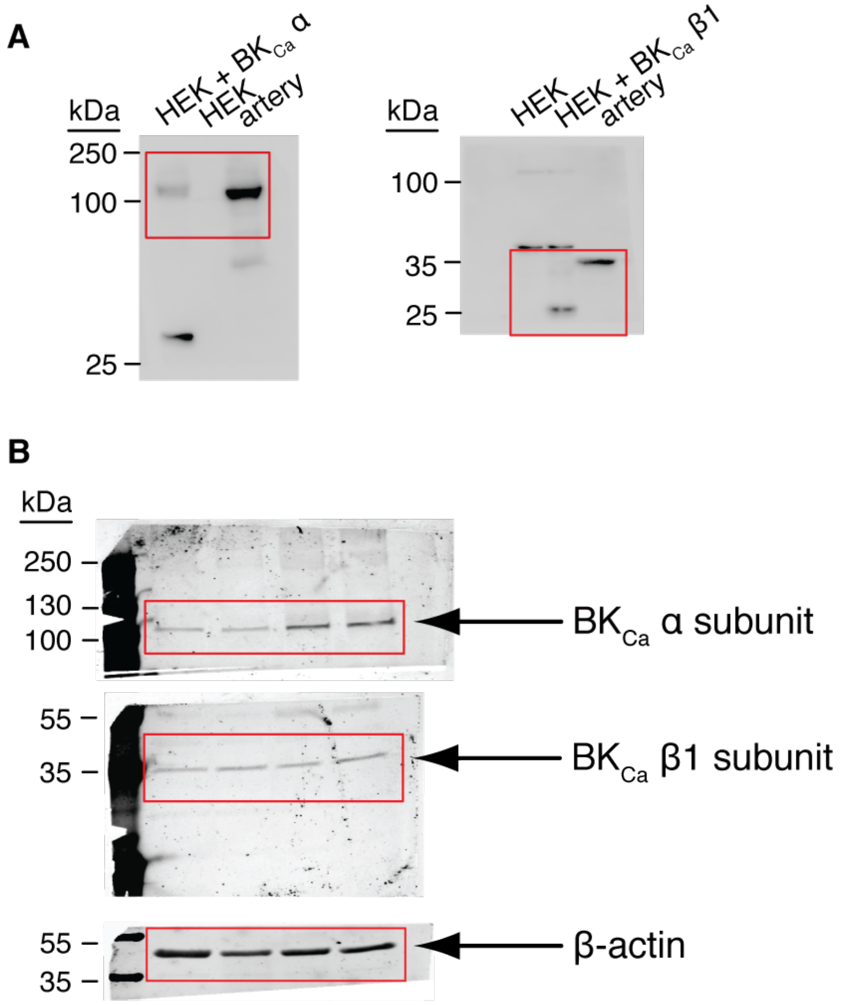

Supplementary Fig. S2: Full-length blots corresponding to A) Figure 6A and B) Figure 6B. Red boxes indicate the crop region displayed in the main figure.

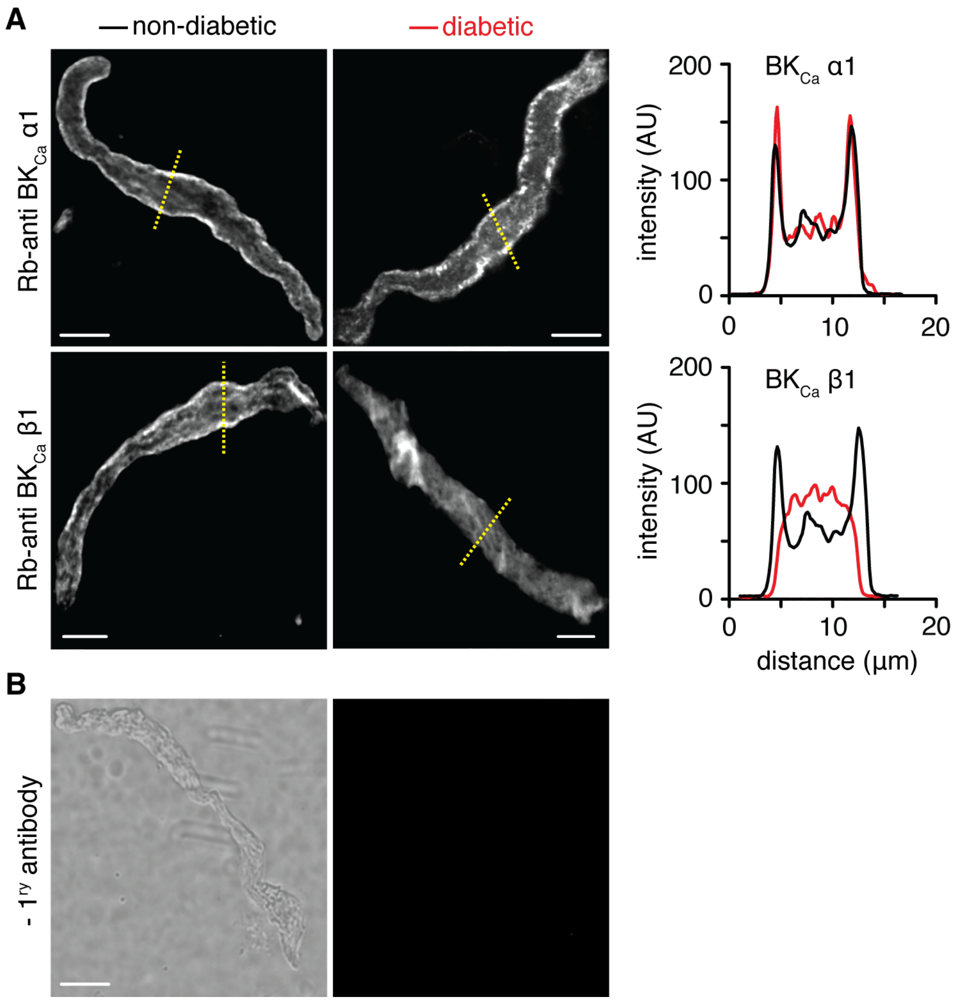

Supplementary Fig. S3: Comparison of the subcellular localization of BK_{Ca} α1 and BK_{Ca} β1 subunit in vascular smooth muscle from obese non-diabetic and diabetic patients. A) Confocal images (*left panels*) and line profile analysis along the dotted yellow lines from the confocal images (*right panels*) of BK_{Ca} α1- and BK_{Ca} β1-associated fluorescence in vascular smooth muscle from non-diabetic and diabetic patients. (n = 10-13 cells per condition. **B)** Exemplary DIC and confocal image of a human vascular smooth muscle in which the primary antibody was excluded from the preparation (e.g. negative control). Scale bar = 10 µm.

**Supplementary Table S1: Available Patients Characteristics**<br>All human arteries and dissociated vascular smooth muscle were obtained from obese non-diabetic and clinically diagnosed diabetic patients undergoing surgical sleeve gastrectomy		
**parameters**	**non-diabetic**	**diabetic**
Sex (female/male)	23 F (88%) / 3 M (12%)	17 F (65%) / 9 M (35%)
age (years)	47.4 ± 2.3	45 ± 2.2
HbA1c (mg/dL)	5.8 ± 0.2	7.1 ± 0.9
HbA1c (mmol/mol)	39.5 ± 2.1	54.0 ± 9.5

Values are mean ± SEM.

**Supplementary Table S2: Characteristics of Arteries in Pressure Myography Experiments**		
	**metabolic state**	
**parameters**	**non-diabetic**	**diabetic**
age (years; # arteries and sex)	47.6 ± 5.1 (3M, 5F)	38.8 ± 3.1 (6F)
baseline diameter (µm)	39.6 ± 6.5	28.5 ± 2.3
myogenic tone (%)	44 ± 8.0	49.3 ± 7.0
diameter of peak IbTx response (µm)	32.3 ± 5.9	27.2 ± 2.2
IbTx-induced constriction (%)	23.2 ± 4.6	4.4 ± 3.5*
diameter of peak 60 K^+ response (µm)	24.3 ± 5.5	12.8 ± 3.0
60 mM K^+-induced constriction (%)	75.0 ± 4.2	71.0 ± 6.3
passive diameter (µm)	73.3 ± 6.4	58.8 ± 4.6

Values are mean ± SEM (* $P < 0.05$; Mann-Whitney test).
